# Antinociceptive Effect of the Essential Oil from *Croton conduplicatus* Kunth (Euphorbiaceae)

**DOI:** 10.3390/molecules22060900

**Published:** 2017-05-30

**Authors:** Raimundo Gonçalves de Oliveira Júnior, Christiane Adrielly Alves Ferraz, Juliane Cabral Silva, Ana Paula de Oliveira, Tâmara Coimbra Diniz, Mariana Gama e Silva, Lucindo José Quintans Júnior, Ana Valéria Vieira de Souza, Uiliane Soares dos Santos, Izabel Cristina Casanova Turatti, Norberto Peporine Lopes, Vitor Prates Lorenzo, Jackson Roberto Guedes da Silva Almeida

**Affiliations:** 1 Center for Studies and Research of Medicinal Plants, Federal University of San Francisco Valley, 56306-385 Petrolina-PE, Brazil; oliveira.farma.junior@gmail.com (R.G.d.O.J.); christiane_adrielly_pe@hotmail.com (C.A.A.F.); larbacjuliane@hotmail.com (J.C.S.); ana_tecquimica@yahoo.com.br (A.P.d.O.); fisiotam7@hotmail.com (T.C.D.); marianagama91@hotmail.com (M.G.e.S.); 2 Departament of Physiology, Federal University of Sergipe, 49100-000 Aracaju-SE, Brazil; lucindojr@gmail.com; 3 Embrapa-Semiárido, 56302-970 Petrolina-PE, Brazil; ana.souza@embrapa.br (A.V.V.d.S.); uilianesoares@hotmail.com (U.S.d.S.); 4 Faculty of Pharmaceutical Sciences of Ribeirão Preto, University of São Paulo, 14040-903 Ribeirão Preto-SP, Brazil; npelopes@gmail.com (I.C.C.T.); npelopes@gmail.com (N.P.L.); 5 Federal Institute of Education, Science and Technology Sertão Pernambucano, 56300-000 Petrolina-PE, Brazil; vitorplorenzo@gmail.com

**Keywords:** pain, medicinal plants, essential oil, terpenoids

## Abstract

Medicinal plants have been widely used in the treatment of chronic pain. In this study, we describe the antinociceptive effect of the essential oil from *Croton conduplicatus* (the EO 25, 50, and 100 mg/kg, i.p.), a medicinal plant native to Brazil. Antinociceptive activity was investigated by measuring the nociception induced by acetic acid, formalin, hot plate and carrageenan. A docking study was performed with the major constituents of the EO (*E*-caryophyllene, caryophyllene oxide, and camphor). The EO reduced nociceptive behavior at all doses tested in the acetic acid-induced nociception test (*p* < 0.05). The same was observed in both phases (neurogenic and inflammatory) of the formalin test. When the hot-plate test was conducted, the EO (50 mg/kg) extended the latency time after 60 min of treatment. The EO also reduced leukocyte migration at all doses, suggesting that its antinociceptive effect involves both central and peripheral mechanisms. Pretreatment with glibenclamide and atropine reversed the antinociceptive effect of the EO on the formalin test, suggesting the involvement of K_ATP_ channels and muscarinic receptors. The docking study revealed a satisfactory interaction profile between the major components of the EO and the different muscarinic receptor subtypes (M2, M3, and M4). These results corroborate the medicinal use of *C. conduplicatus* in folk medicine.

## 1. Introduction

Pain is a complex experience involving noxious stimuli and emotional processing. It is a pathological consequence of various disorders, but which has a protective role representing, in many cases, the only symptom for the diagnosis of diseases [1]. In contrast, when persistent, pain causes negative emotional and physiological reactions, leaving the patient in a debilitating state. Epidemiological studies suggest that the world prevalence of chronic pain is approximately 40% [2,3,4,5] and its severity usually correlates with a decreasing in physical and mental health [6].

Pain commonly involves central and peripheral mechanisms, which makes it very difficult to choose an appropriated pharmacological therapy. Although highly effective, central analgesics are usually not disassociated with important adverse effects, such as dependence, respiratory depression, and constipation [7,8]. Furthermore, peripheral analgesics also have undesirable effects, such as gastrointestinal and renal lesions [9,10]. In this context, research groups have been dedicated to discover new molecules with analgesic potential and low toxicity. In fact, several pharmacological studies involving medicinal plants and their essential oils have been performed for this purpose [11,12,13,14].

*Croton conduplicatus* Kunth. (Euphorbiaceae) is a Brazilian medicinal plant, endemic of the Caatinga biome and popularly known as “quebra-faca” [15]. Its leaves and stem-bark have a strong and characteristic odor, being considered an aromatic plant. In folk medicine, this plant is used as a natural analgesic for the treatment of headache and stomach disorders [16]. However, to date, there are no reports in the literature that can scientifically validate the popular use of this species. Therefore, this paper describes the antinociceptive effect of the essential oil from *C. conduplicatus* (the EO) and possible mechanisms involved, aided by a docking study of its chemical constituents.

## 2. Results

### 2.1. Chemical Composition of the EO

*Croton conduplicatus* produced a colorless essential oil with characteristic odor. GC-MS analysis showed the presence of 48 distinct peaks, of which 38 were identified, corresponding to 90.5% of its entire chemical composition. The sesquiterpenes (*E*)-caryophyllene (13.72%), and caryophyllene oxide (13.15%) and the monoterpene camphor (8.25%) were considered as the major constituents of the EO (Table 1).

### 2.2. Acetic-Acid-Writhing-Induced Nociception

**Figure 1:**
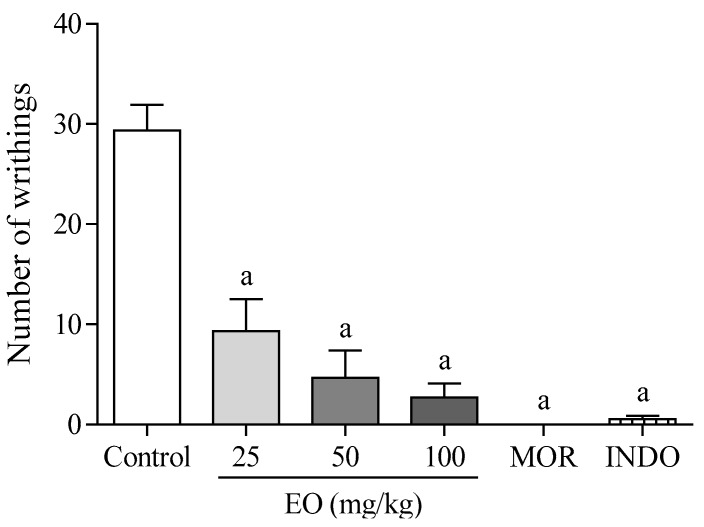
The effect of the essential oil of *C. conduplicatus* (the EO 25, 50, and 100 mg/kg, i.p.), morphine (MOR 10 mg/kg, i.p.) and indomethacine (20 mg/kg, i.p.) in the acetic-acid-writhing-induced nociception test, in mice (*n* = 6, per group). Values are expressed as mean ± SEM, where a indicates *p* < 0.05, significantly different from the control group, according to ANOVA, followed by Tukey′s test.

Figure 1 shows that the EO produced a significant (*p* < 0.05) the antinociceptive effect in mice during acetic acid writhing induced nociception test. The EO at doses of 25, 50, and 100 mg/kg (i.p.) presented 68, 84, and 91% of antinociceptive activity, respectively. Morphine and indomethacin reduced the number of writhings by 100 and 98%, respectively.

### 2.3. Formalin-Induced Nociception

**Figure 2:**
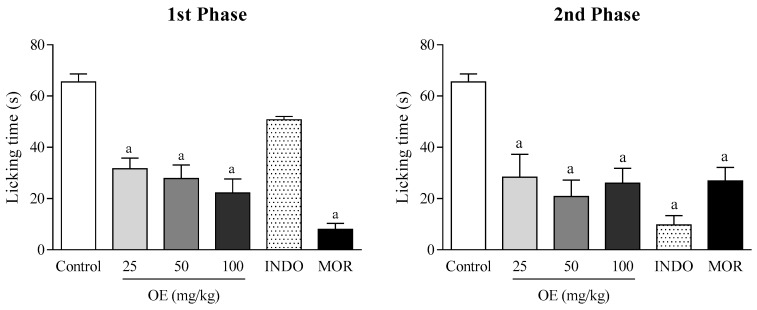
The effect of the essential oil of *C. conduplicatus* (the EO 25, 50, and 100 mg/kg, i.p.), morphine (MOR 10 mg/kg, i.p.) and indomethacine (20 mg/kg, i.p.) in the formalin-induced nociception test, in mice (*n* = 6, per group). Values are expressed as mean ± SEM, where a indicates *p* < 0.05, significantly different from the control group, according to ANOVA, followed by Tukey′s test.

In the formalin-induced nociception model, the EO treatment significantly reduced the paw licking time compared to the control group, at all doses used, and in both phases of the test (*p* < 0.05). In the first phase, the EO (25, 50, and 100 mg/kg) showed 52, 57 and 66% of antinociceptive activity, respectively, whereas in the second phase, the effect varied between 71 and 79%. In addition, indomethacin was effective only in the second phase of the test, whereas morphine demonstrated satisfactory antinociceptive activity in both phases (Figure 2).

#### 2.3.1. Involvement of Muscarinic Receptors

**Figure 3:**
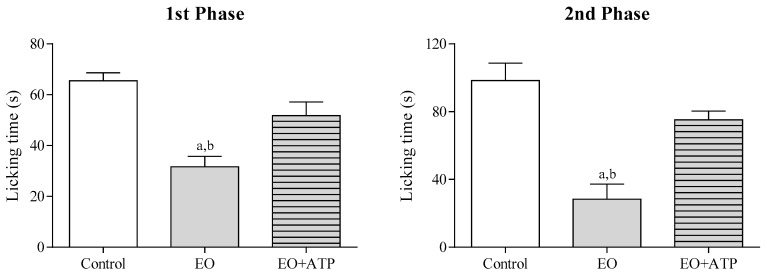
The effect of the essential oil of *C. conduplicatus* (the EO 25 mg/kg, i.p.) and the EO + ATP (25 and 0.1 mg/kg, respectively, i.p.) in the first and second phase of formalin-induced nociception test (*n* = 6, per group). Values are expressed as mean ± SEM, where a and b indicate *p* < 0.05, significantly different from control and the EO + ATP groups, respectively, according to ANOVA, followed by Tukey’s test.

To evaluate the involvement of muscarinic receptors in the antinociceptive effect of the EO, animals were pretreated with atropine (ATP 0.1 mg/kg, i.p.) during the formalin test. Figure 3 shows that atropine pretreatment reversed the antinociceptive effect of the EO (25 mg/kg, i.p.) in both phases of the test (*p* < 0.05), suggesting that the cholinergic system may be involved.

#### 2.3.2. Involvement of ATP-Sensitive K^+^ Channels

**Figure 4:**
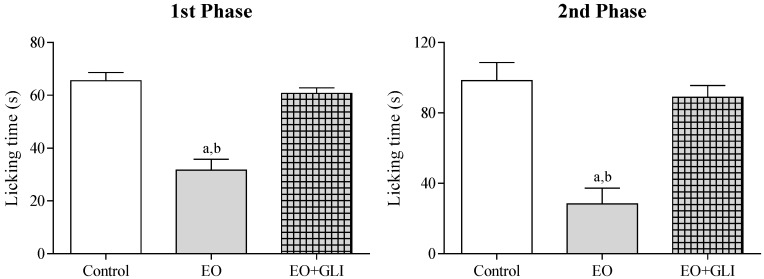
The effect of the essential oil of *C. conduplicatus* (the EO 25 mg/kg, i.p.) and the EO + GLI (25 and 2 mg/kg, respectively, i.p.) in the first and second phase of formalin-induced nociception test (*n* = 6, per group). Values are expressed as mean ± SEM, where a and b indicate *p* < 0.05, significantly different from control and the EO + GLI groups, respectively, according to ANOVA, followed by Tukey′s test.

Figure 4 shows the involvement of ATP-sensitive K^+^ channels in the antinociceptive effect of the EO. Glibenclamide pretreatment (GLI 2 mg/kg, i.p.) significantly (*p* < 0.05) reversed the antinociceptive activity of the EO (25 mg/kg, i.p.) in both phases of the formalin induced nociception test.

#### 2.3.3. Involvement of Opioid Receptors

**Figure 5:**
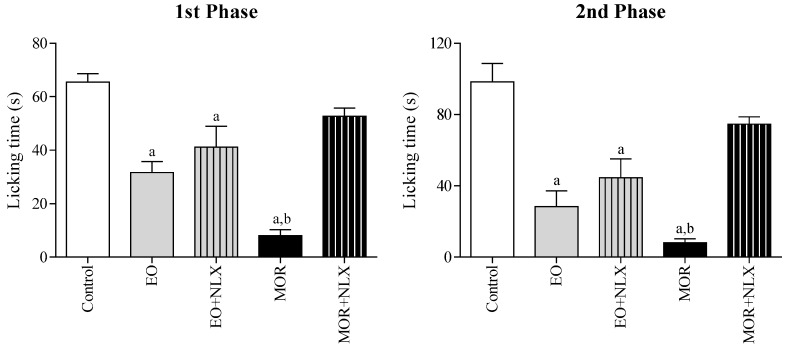
The effect of the essential oil of *C. conduplicatus* (the EO 25 mg/kg, i.p.), the EO + NLX (25 and 1.5 mg/kg, respectively, i.p.), morphine (MOR 10 mg/kg, i.p.) and MOR + NLX (10 and 1.5 mg/kg, respectively, i.p.) in the first and second phase of formalin-induced nociception test (*n* = 6, per group). Values are expressed as mean ± SEM, where a and b indicate *p* < 0.05, significantly different from control and MOR + NLX groups, respectively, according to ANOVA, followed by Tukey′s test.

To evaluate the involvement of opioid receptors, mice were pretreated with naloxone (NLX 1.5 mg/kg, i.p.) in the formalin test. This pretreatment did not promote any significant change in the antinociceptive effect of the EO (Figure 5). In contrast, naloxone significantly reversed (*p* < 0.05) the antinociceptive effect of morphine (10 mg/kg, i.p.) in both phases of the test. This result suggests that the EO acts differently from morphine, without directly involving opioid receptors.

### 2.4. Hot Plate Test

**Figure 6:**
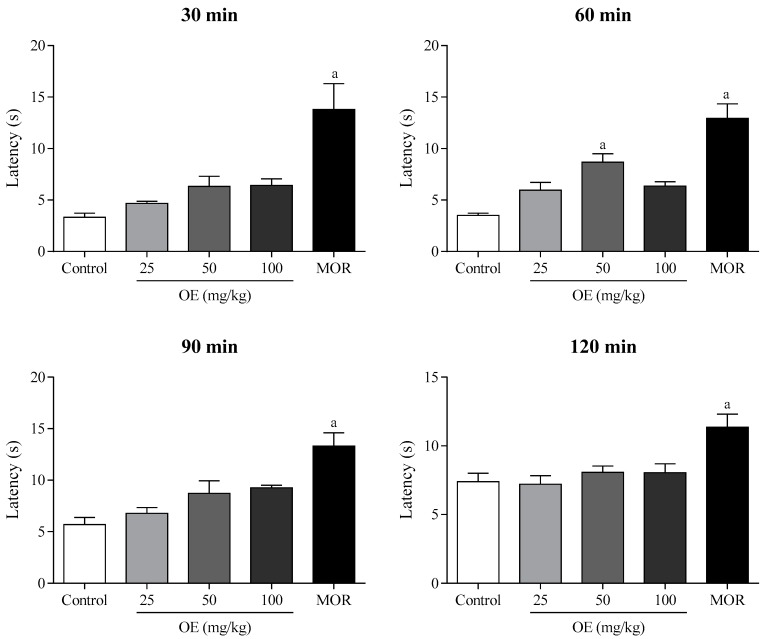
The effect of the essential oil of *C. conduplicatus* (the EO 25, 50, and 100 mg/kg, i.p.) and morphine (MOR 10 mg/kg, i.p.) in the hot plate test (*n* = 6, per group). Values are expressed as mean ± SEM, where a indicates *p* < 0.05, significantly different from control group, according to ANOVA, followed by Tukey′s test.

In the hot plate test, the EO (50 mg/kg, i.p.) increased the latency time of the animals compared to the control group (*p* < 0.05), after 60 min of treatment, suggesting a possible antinociceptive activity mediated by central mechanisms (Figure 6). Morphine (10 mg/kg, i.p.) showed satisfactory antinociceptive effect during all the test (30–120 min).

### 2.5. Leukocyte Migration to the Peritoneal Cavity

**Figure 7:**
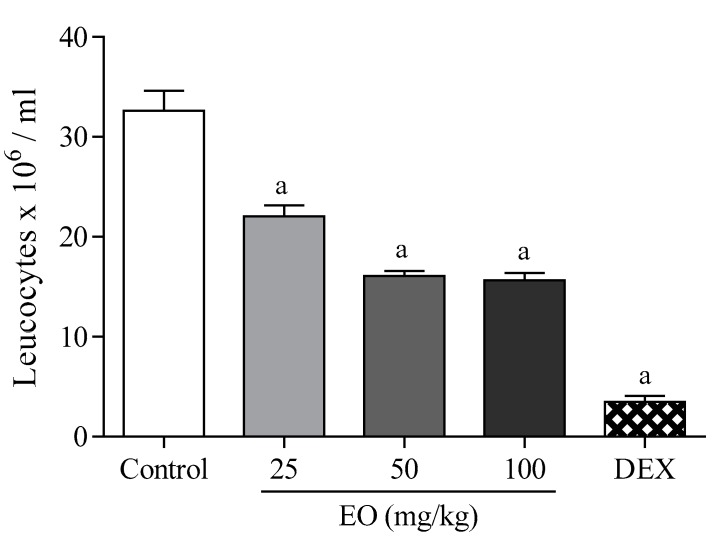
The effect of the essential oil of *C. conduplicatus* (the EO 25, 50, and 100 mg/kg, i.p.) and dexamethasone (DEX 2 mg/kg, i.p.) in the leukocyte migration to the peritoneal cavity test (*n* = 6, per group). Values are expressed as mean ± SEM, where a indicates *p* < 0.05, significantly different from control group, according to ANOVA, followed by Tukey′s test.

In this model, the EO (25, 50, and 100 mg/kg, i.p.) significantly decreased (*p* < 0.05) the migration of leukocytes to the peritoneal region at all doses (32–52%), indicating that the antinociceptive effect of the essential oil also involves peripheral mechanisms (Figure 7). Dexamethasone (2 mg/kg, i.p.) also had a significant pharmacological effect compared to the control group (89%).

### 2.6. Rota-Rod Test

**Figure 8:**
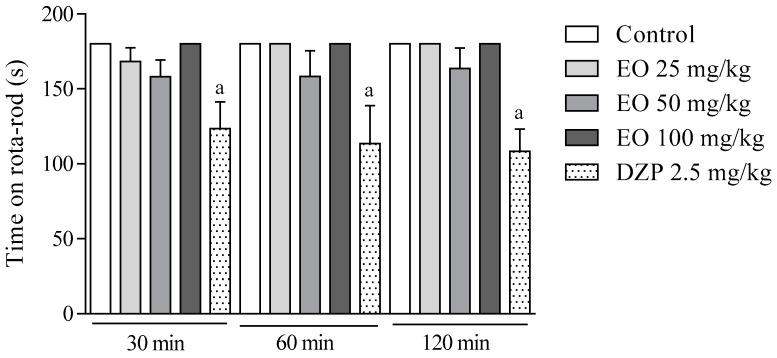
The effect of the essential oil of *C. conduplicatus* (the EO 25, 50, and 100 mg/kg, i.p.) and diazepam (DZP 2.5 mg/kg, i.p.) in the rota-rod test (*n* = 6, per group). Values are expressed as mean ± SEM, where a indicates *p* < 0.05, significantly different from negative control group, according to ANOVA, followed by Tukey′s test.

The effect of the EO on the motor coordination was investigated in the rota-rod test. Animals treated with the EO (25, 50, and 100 mg/kg, i.p.) did not present any significant changes in comparison to the control group (Figure 8). In contrast, diazepam (2.5 mg/kg, i.p.) decreased (*p* < 0.05) the residence time of the animals on the rota-rod during all analysis time (30–120 min).

### 2.7. Docking Study

**Figure 9:**
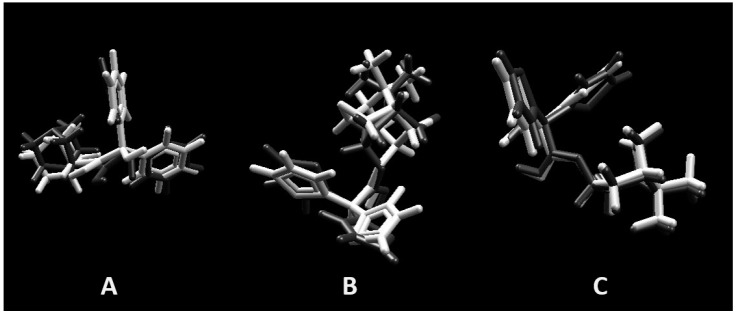
Superposition of crystal pose (white) and docking pose (gray) validating the methodology. (**A**) Muscarinic receptors M2 (RMSD = 0.6043A°); (**B**) muscarinic receptors M3 (RMSD = 1.0351A°); and (**C**) muscarinic receptors M4 (RMSD = 0.3607A°).

Docking was validated redocking the originals ligands in active site of muscarinic receptors M2, M3, and M4 as observed in crystallography pdb file. The same parameter described to evaluate majoritary compounds of the the EO (camphor, caryophyllene oxide, and (*E*)-caryophyllene) was used in validation. Superpositions of poses are represented in Figure 9 and shows a good match, with root mean square deviation (RMSD) values of 0.6043A°, 1.0351A°, and 0.3607A° for muscarinic receptors M2, M3, and M4, respectively.

The structure of ligands (3*R*)-1-azabicyclo[2.2.2]oct-3-yl hydroxy(diphenyl)acetate [(*R*)-(−)-3-Quinuclidinyl benzilate (QNB)] co-crystalized with muscarinic receptor M2 and (1*R*,2*R*,4*S*,5*S*,7*S*)-7-{[hydroxy(dithiophen-2-yl)acetyl]oxy}-9,9-dimethyl-3-oxa-9-azoniatricyclo[3.3.1.0~2,4~]nonane (tiotropium) co-crystalized with muscarinic receptor M3 and M4, were used in docking performance for energy evaluation. Binding energies of camphor, caryophyllene oxide, and (*E*)-caryophyllene, as well as co-crystalized ligands are show in Table 2.

## 3. Discussion

GC-MS analysis showed two sesquiterpenes ((*E*)-caryophyllene 13.72%, and caryophyllene oxide 13.15%) and one monoterpene (camphor 8.25%) as the major chemical constituents of the EO (Table 1). Previous studies have indicated α-pinene (25.84–35.35%) and β-pinene (7.79–16.77%) as the major constituents of the essential oil extracted from the stem-barks of *C. conduplicatus*, suggesting that season may interfere with the chemical composition of the EO. However, camphor (6.37–9.32%) and (*E*)-caryophyllene (4.66–7.80%) also appear among the main constituents of the essential oil [17]. Studies performed with caryophyllene exhibited antinociceptive activity by mechanisms that may involve peripheral cannabinoid and opioid receptors [18]. Caryophyllene oxide showed antinociceptive activity, which is probably related to central and peripheral analgesia, along with anti-inflammatory activity [19].

The EO presented a strong antinociceptive effect in the acetic acid-induced nociception test, significantly reducing the number of writhing in the animals (Figure 1). However, this is a rather specific experimental model for evaluating the antinociceptive effect of drugs. The acetic acid intraperitoneal administration promotes the release of several mediators involved in both central (neurogenic) and peripheral (inflammatory) mechanisms of pain, and it is not possible to distinguish in what way the EO would be acting [20]. For this reason, the formalin-induced nociception test was performed.

In the formalin-induced nociception test, the EO reduced paw-licking time in both phases (Figure 2). Behavioral responses to formalin injection follow a biphasic pattern composed of an acute early phase (first phase) and a longer (second phase). The first phase is related to direct chemical stimulation of nociceptors distributed in the afferent C fibers and, in part, in the Aδ fibers. This phase is marked by the release of excitatory amino acids, nitric oxide and substance P. Meanwhile, the second phase involves the release of several pro-inflammatory mediators, such as bradykinin, histamine, nitric oxide, and prostaglandins [21]. Having presented an antinociceptive effect in both phases, the EO may be acting by central or peripheral mechanisms. To confirm these hypotheses, the hot-plate and leukocyte migration tests were performed.

The EO (50 mg/kg) increased the latency time on the hot-plate 60 min after its administration (Figure 6). In addition, the EO reduced leukocyte migration at all doses tested (Figure 7). These data corroborate the results found in the formalin test, suggesting that the antinociceptive effect of the EO may involve neurogenic and inflammatory responses. In this context, the formalin test was selected to evaluate the mechanisms of action involved in the pharmacological effect of the EO.

Pre-treatment with naloxone did not reverse the antinociceptive activity of the EO (Figure 5), indicating that opioid receptors are not involved in this effect. However, glibenclamide and atropine completely reversed the antinociceptive effect of the EO, suggesting the involvement of ATP-sensitive K^+^ channels (K_ATP_) and muscarinic receptors, respectively (Figure 3 and Figure 4). It has been shown that many types of potassium channels, including K_ATP_ channels, regulate nociception [22,23]. Similarly, muscarinic receptors have been studied as a therapeutic target in the treatment of central and peripheral pain [24,25]. In fact, muscarinic receptors (mAChR) have been considered an important molecular target in the development of new analgesic drugs due to their role in the regulation of chronic pain. Most pharmacological studies indicate that peripheral and central antinociception is mediated primarily by the M2, M3 and M4 mAChR subtypes. These subtypes are present in the spinal cord and therefore play an important role in nociception mechanisms [25,26]. In this sense, we also decided to evaluate the interaction profile of the main chemical constituents of the EO in different subtypes of muscarinic receptors (M2, M3 and M4) through a docking study.

Evaluating binding energy values of three majority terpenes from the EO, (*E*)-caryophyllene (13.72% GC-MS) and caryophyllene oxide (13.15% GC-MS) present better performance in comparison with camphor (8.25% GC-MS), contributing for biological activity observed once (*E*)-caryophyllene and caryophyllene oxide represent more than a quarter of the EO composition. Although, caryophyllene oxide show better energy values between terpenes evaluated through in silico approach, its value still lower than QNB and tiotropium, ligands used as standard. However, binding energy values allow suggest a synergy effect specially between (*E*)-caryophyllene and caryophyllene oxide (Table 2).

Finally, the effect of the EO on the motor coordination of the animals was evaluated through the rota-rod test (Figure 8). In this experimental model, no significant change was observed, suggesting that the EO exerts its antinociceptive effect, but without interfering in the motor coordination of the animals.

## 4. Materials and Methods

### 4.1. Plant Material

The stem-barks of *Croton conduplicatus* Kunth (Euphorbiaceae) were collected from a single adult individual in the city of Petrolina (Coordinates: S 09°03′54′′; W 40°19′12′′), State of Pernambuco, Brazil, in September of 2012. A voucher specimen (HTSA2421) was deposited at the Herbário do Trópico Semiárido (HTSA) of the Empresa Brasileira de Pesquisa Agropecuária do Semiárido (EMBRAPA-Semiárido).

### 4.2. Essential Oil Extraction

Stem-bark (100 g) were submitted to extraction by hydrodistillation in a Clevenger-type apparatus for 2 h. The essential oil (the EO) obtained (1.50 mL) was dried with anhydrous sodium sulfate and filtered, and then stored in a refrigerator at a temperature of 4 °C until the pharmacological tests and chemical analysis.

### 4.3. Chemical Analysis of the Essential Oil (the EO)

The compounds in the EO were investigated on a Shimadzu QP-2010 (Shimadzu Corporation, Kyoto, Japan) gas chromatograph coupled to a mass spectrometer (GC-MS). The following conditions were used: DB-5MS column Agilent Technologies (30 m × 0.25 mm × 0.25 μm); helium (99.999%) carrier gas at a constant flow of 1.1 mL/min; injection volume of 1.0 μL; injector split ratio of 1:10; injector temperature of 250 °C; electron impact mode at 70 eV; ion-source temperature of 280 °C and transfer line temperature of 260 °C. The oven temperature was programmed from 60 °C, with an increase of 3 °C/min to 240 °C. A mixture of linear hydrocarbons (C_8_H_18_–C_20_H_42_) was injected under the same experimental conditions. The identification of the constituents in the EO was performed by comparing the spectra obtained with those of the equipment database (Wiley 7 lib and Nist 08 lib) and by using the Retention Index (RI), calculated for each constituent as previously described [27,28]. The data were acquired and processed on a PC with Shimadzu GC-MS Solution software (Shimadzu Corporation, Kyoto, Japan).

### 4.4. Animals

Adult male Swiss mice (30–40 g) were randomly housed in appropriate cages at 22 ± 2 °C on a 12 h light/dark cycle (lights on at 7:00 a.m.) with free access to food and water. Animals were allowed to have a period of acclimation (24 h) before any pharmacological test. They were used in groups of six animals each (*n* = 6). All tests were performed by the same visual observers. All experimental protocols and procedures were approved by the Animal Care and Use Committee from Federal University of San Francisco Valley by number 0004/140814.

### 4.5. Drug Treatments 

Animals (*n* = 6) were submitted to different treatments, all intraperitoneally (i.p.). The control group was treated with the vehicle used for the EO and standard drugs solubilization (0.9% saline + 10 µL of Tween 80, 10 mL kg^−1^). Positive control groups were treated with morphine (10 mg/kg), indomethacin (20 mg/kg), diazepam (2.5 mg/kg), or dexamethasone (2 mg/kg), according to protocol. The other groups were treated with the EO at doses of 25, 50, and 100 mg/kg in all pharmacological tests. Glibenclamide (2 mg/kg), naloxone (1.5 mg/kg) and atropine (0.1 mg/kg) were used to evaluate possible mechanisms of action.

### 4.6. Acetic-Acid-Writhing-Induced Nociception

This test was performed according to the protocol previously described [29], with some adaptations [30]. Acetic acid 0.9% solution was prepared in saline and administered intraperitoneally to all animals (0.1 mL/10g). Groups of six mice were treated with vehicle (control), morphine (10 mg/kg), indomethacin (20 mg/kg), and the EO (25, 50, and 100 mg/kg) 30 min before the acetic acid injection. The number of writhings occurring between 5 and 15 min after acetic acid injection was counted to each group. Writhing was defined as a contraction of the abdominal wall, pelvic rotation, followed by hind limb extension.

### 4.7. Formalin-Induced Nociception

A formalin 2.5% solution (in saline) was injected into the right hind paw of the mice (20 μL/paw subplantar). Vehicle (control group), morphine (10 mg/kg), indomethacin (20 mg/kg), and the EO (25, 50, and 100 mg/kg) were administered 60 min before the formalin injection [30]. Animals were observed in chambers with a mirror mounted on three sides and the time (s) spent licking and biting the injected paw was measured as a nociceptive response. Responses were measured for 5 min after formalin injection (first phase, neurogenic) and 15–30 min after formalin injection (second phase, inflammatory). Mice were pretreated with glibenclamide (2 mg/kg), naloxone (1.5 mg/kg), and atropine (0.1 mg/kg) 15 min before administration of morphine (10 mg/kg) or the EO (25 mg/kg) to evaluate the involvement of ATP-sensitive K^+^ channels, opioid, and muscarinic receptors. After 60 min, the animals were observed under the same conditions.

### 4.8. Hot Plate Test

This experimental model was performed to investigate the antinociceptive effect of the EO by central mechanisms, applying thermal stimulation. Mice were placed on hot plate apparatus (Insight, Wembley, UK) at 55 ± 0.5 °C. A pre-selection of the animals was accomplished 24 h before the experiments. Animals showing a reaction time (defined as the latency for licking the hind feet or jumping) greater than 20 s were discarded. Selected animals were treated with vehicle (control group), morphine (10 mg/kg) and the EO (25, 50 and 100 mg/kg). Latency time was recorded for each animal on the hot plate (55 °C) during a maximum period of 20 s, at intervals of 30, 60, 90, and 120 min after treatments [31].

### 4.9. Leukocyte Migration to the Peritoneal Cavity

This test was performed to evaluate the antinociceptive activity of the EO by peripheral mechanisms (possible anti-inflammatory effect) [32]. A carrageenan 1% solution (0.25 mL per animal, i.p.) was used to induce the migration of leukocytes into the intraperitoneal cavity. Mice were treated with the EO (25, 50, and 100 mg/kg) and dexamethasone (2 mg/kg) 30 min after carrageenan injection. Leukocyte migration was assessed 4 h post-challenge. Animals were euthanized and cells from the peritoneal cavity were collected with 3 mL of saline containing 1 mM EDTA. Subsequently, the abdominal cavity of the animals was massaged for 15 seconds and the peritoneal fluid was collected and centrifuged (3000 rpm, for 5 min) at room temperature. The supernatant was discarded and 300 μL of EDTA was added to the precipitate, from which an aliquot of 10 μL was removed. Two-hundred microliters (200 μL) of Turk's solution was added to the aliquot and the total cells were counted under an optical microscope. The results were expressed as number of leukocytes × 10^6^/mL.

### 4.10. Rota-Rod Test

A rota-rod apparatus (Insight, Wembley, Brazil) was used for the evaluation of motor coordination [32]. Initially, animals capable of remaining on the rota-rod apparatus longer than 180 s (7 rpm) were selected 24 h before the test. Groups of six mice were treated with vehicle (control group), the EO (25, 50 and 100 mg/kg), and diazepam (2.5 mg/kg). Each animal was individually evaluated on the rota-rod apparatus at 30, 60, and 120 min after treatments, and the time (s) spent on top of the bar was recorded for up to 180 s.

### 4.11. Docking Study

The structure of Muscarinic receptors M2 in complex with (3*R*)-1-azabicyclo[2.2.2]oct-3-yl hydroxy(diphenyl)acetate (PDB ID 3UON) [33], M3 in complex with tiotropium (PDB ID 4DAJ) [34], and M4 in complex with tiotropium (PDB ID 5DSG) [35] were downloaded from the Protein Data Bank (http://www.rcsb.org/pdb/home/home.do). The structure of the majority compounds camphor, caryophyllene oxide, and (*E*)-caryophyllene were submitted to molecular docking using the Molegro Virtual Docker, v. 6.0.1 (MVD) [36]. All of the water compounds were deleted from the enzyme structure, and the enzyme and compound structures were prepared using the same default parameter settings in the same software package (Score function: MolDock Score; ligand evaluation: internal ES, internal HBond, Sp2-Sp2 torsions, all checked; number of runs: 10 runs; algorithm: MolDock SE; maximum interactions: 1500; maximum population size: 50; maximum steps: 300; neighbor distance factor: 1.00; maximum number of poses returned: 5). The docking procedure was performed using a grid 15 Å in radius and 0.30 in resolution to cover the ligand-biding site of the GABAR structure. The Moldock score [grid] algorithm was used as the score function, and the Moldock search algorithm was used [36]. Docking was validated by redocking the original ligand on the biding site and calculating the RMSD values.

### 4.12. Statistical Analysis

All data obtained were presented as mean ± standard error of the mean (SEM) and the statistical significance was determined using an analysis of variance (ANOVA) followed by Tukey′s test. Values were considered significantly different when *p* < 0.05. All analysis was performed with GraphPad Prism 6.0 software (Graph Pad Prism Software Inc., San Diego, CA, USA).

## 5. Conclusions

This study described, for the first time, the antinociceptive effect of the essential oil of the stem-barks from *C. conduplicatus* (the EO), an aromatic plant native to Brazil, used as a natural analgesic. In addition to support the popular use of this species, the results indicated that the EO acts through central and peripheral mechanisms, possibly involving K_ATP_ channels and muscarinic receptors.

## Figures and Tables

**Table 1:** Chemical constituents of the essential oil from stem bark of *Croton conduplicatus* Kunth. (Euphorbiaceae).

Peak	RT (min)	RI	Compound	% GC-MS
1	8.44	914	α-Thujene	0.04
2	8.91	926	α-Pinene	5.57
3	9.45	941	Camphene	0.97
4	10.56	969	β-Pinene	4.88
5	12.76	1026	1,8-Cineole	2.56
6	14.54	1072	*cis*-Linalool oxide	0.31
7	15.02	1085	NI	0.22
8	15.13	1088	*trans*-Linalool oxide	1.20
9	15.74	1104	Linalool	0.74
10	15.81	1106	NI	0.72
11	16.04	1112	*exo*-Fenchol	0.56
12	16.97	1137	*cis*-Pinocarveol	0.51
13	17.09	1140	Camphor	8.25
14	17.28	1145	Camphene hydrate	0.36
15	17.84	1161	Pinocarvone	0.16
16	17.99	1165	Borneol	1.29
17	18.48	1178	Terpinen-4-ol	1.05
18	19.08	1194	α-Terpineol	0.62
19	19.11	1195	Myrtenal	0.89
20	20.55	1236	Thymol, methyl ether	0.34
21	24.79	1362	Cyclosativene	0.33
22	25.17	1373	α-Copaene	0.97
23	25.91	1396	α-Gurjunene	0.39
24	26.55	1417	(*E*)-Caryophyllene	13.72
25	26.87	1427	NI	0.43
26	27.62	1451	α-Humulene	6.05
27	27.84	1458	*Allo*-aromadendrene	0.23
28	28.07	1465	9-epi-(*E*)-Caryophyllene	0.19
29	28.36	1475	Germacrene D	4.01
30	28.64	1484	NI	0.31
31	29.10	1498	α-Muurolene	3.66
32	29.38	1508	β-Bisabolene	0.26
33	29.50	1512	γ-Cadinene	0.17
34	29.79	1522	δ-Cadinene	0.65
35	30.23	1537	NI	0.39
36	30.61	1550	Elemol	0.81
37	31.52	1581	Caryophyllene oxide	13.15
38	31.99	1597	Guaiol	2.51
39	32.10	1601	Rosifoliol	0.83
40	32.28	1607	Humulene epoxide II	4.19
41	32.44	1613	Eudesmol	1.94
42	32.78	1625	Muurolol	3.17
43	33.01	1633	NI	3.04
44	33.11	1637	NI	1.83
45	33.48	1650	Agarospirol	3.01
46	33.74	1660	NI	1.19
47	33.90	1665	NI	0.97
48	40.62	1926	NI	0.35
Total identified				90.55

RT: retention time of compounds. RI: retention indices on DB-5MS column (relative to *n*-alkanes). NI: not identified compound.

**Table 2:** MolDock Score energies of the majority of the compounds of the EO and original ligands on muscarinic receptors M2 (3UON), M3 (4DAJ), and M4 (5DSG).

Compounds	3UON	4DAJ	5DSG
Camphor	−70.79	−68.33	−75.02
Caryophyllene oxide	−110.91	−114.83	−119.26
(*E*)-Caryophyllene	−99.35	−101.76	−105.77
QNB	−49.49	−146.61	−146.30
Tiotropium	–162.89	−167.60	−177.81

## Supplementary Materials

Supplementary File 1
