# Supplementary material for: Antinociceptive Effect of the Essential Oil from Croton conduplicatus Kunth (Euphorbiaceae)
Source: Molecules. 2017 May 30;22(6):900. doi: 10.3390/molecules22060900 (PMC6152674; doi:10.3390/molecules22060900)
Supplement: Supplementary File 1 [file molecules-22-00900-s001.pdf]

Supplementary Material - Mass spectra of unidentified components in the essential oil of *Croton conduplicatus* Kunth.

Peak 7

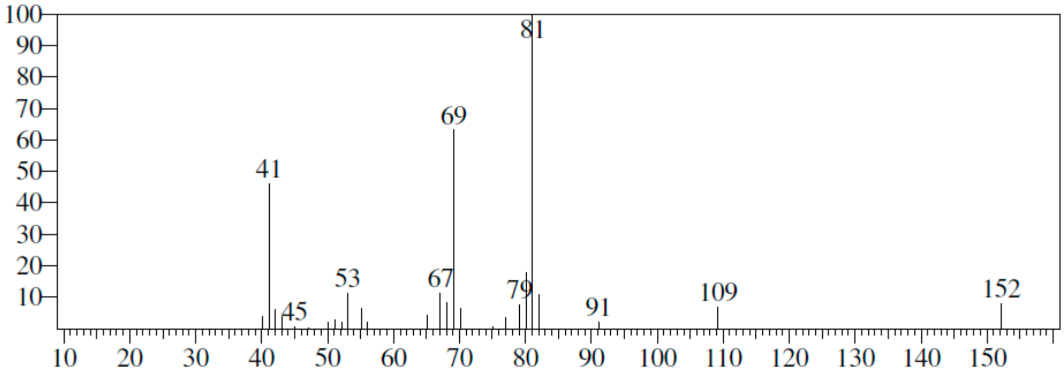

Peak 10

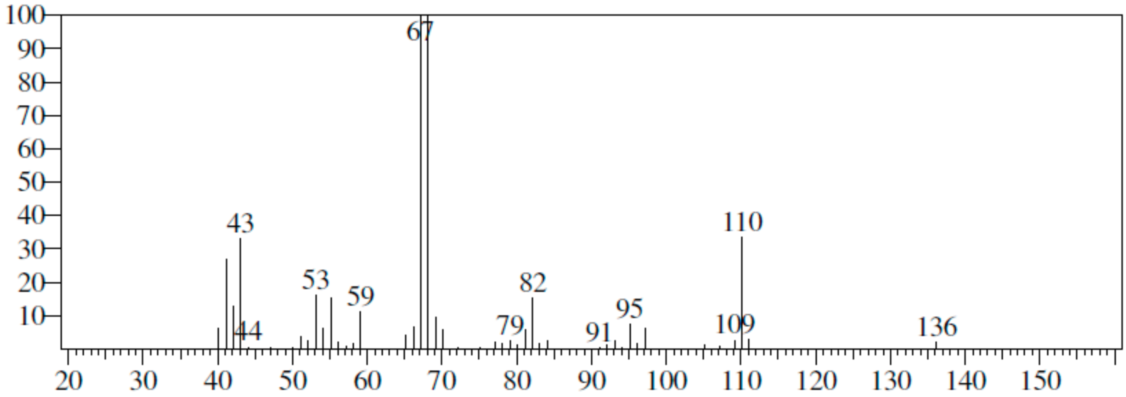

Peak 25

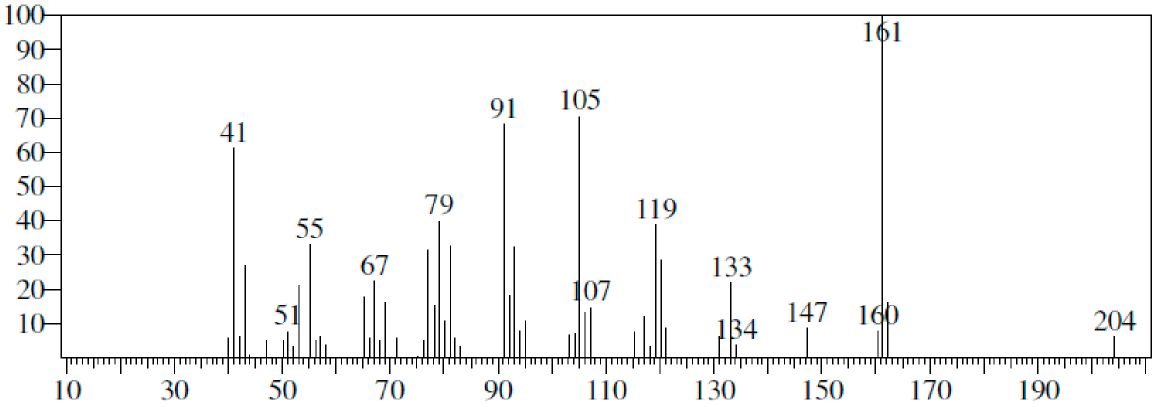

Peak 30

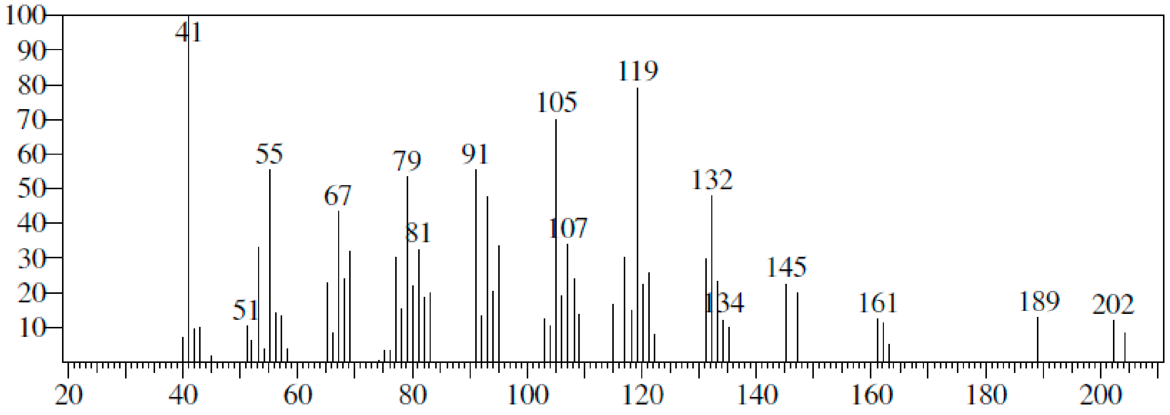

Peak 35

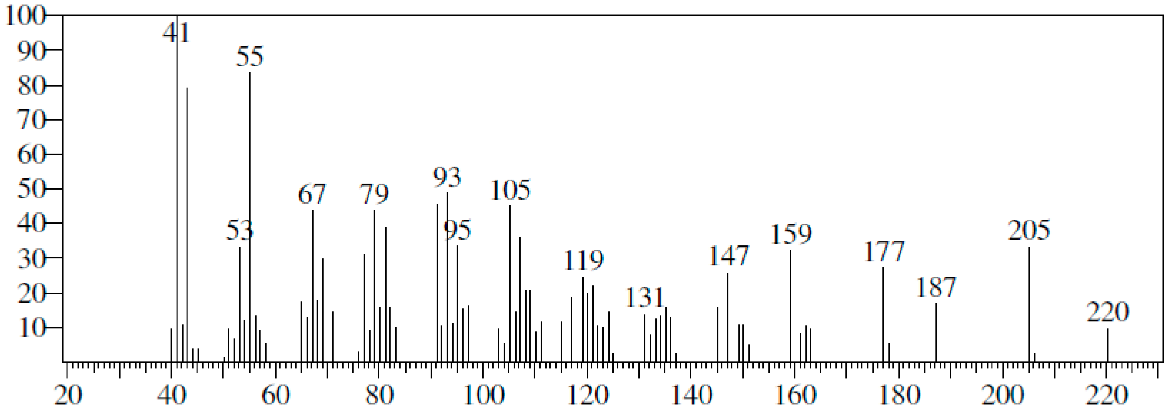

Peak 43

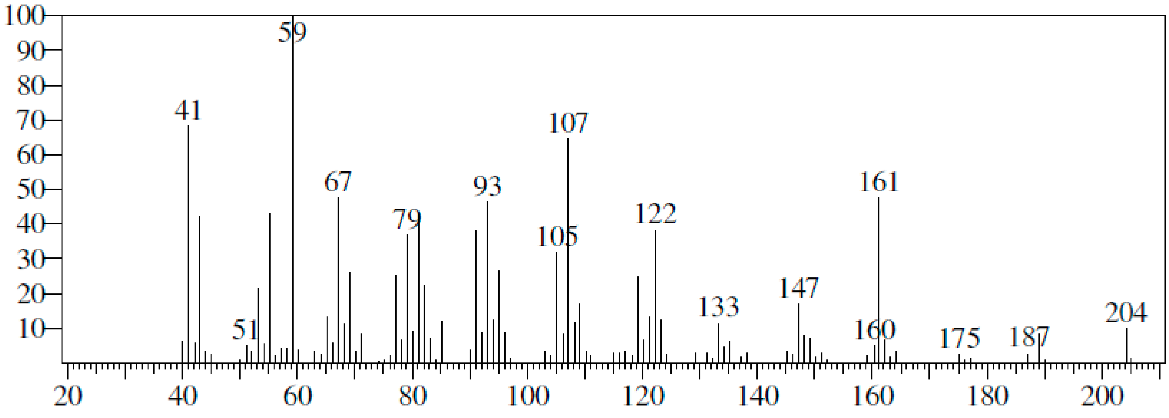

Peak 44

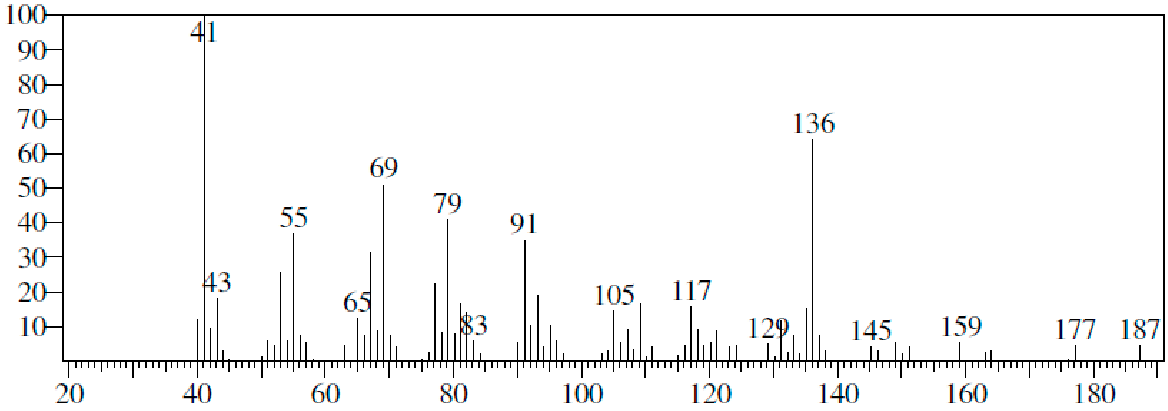

Peak 46

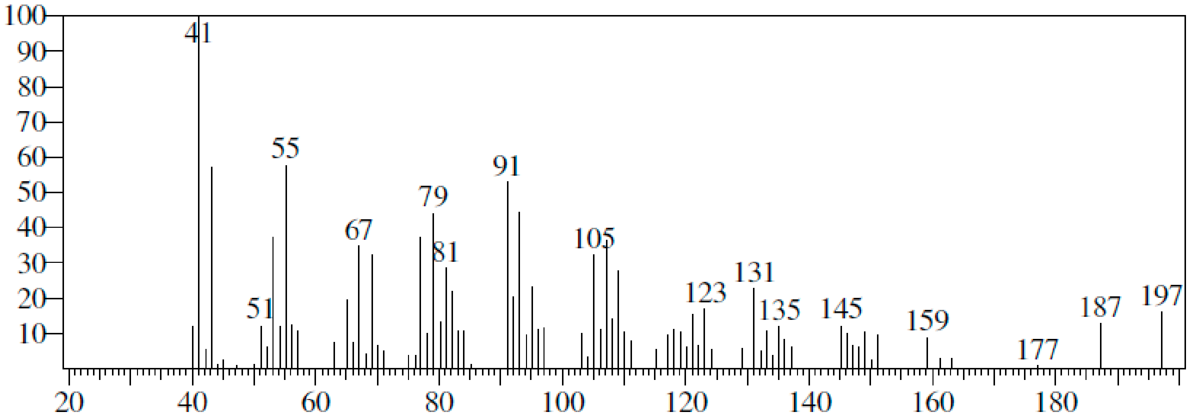

Peak 47

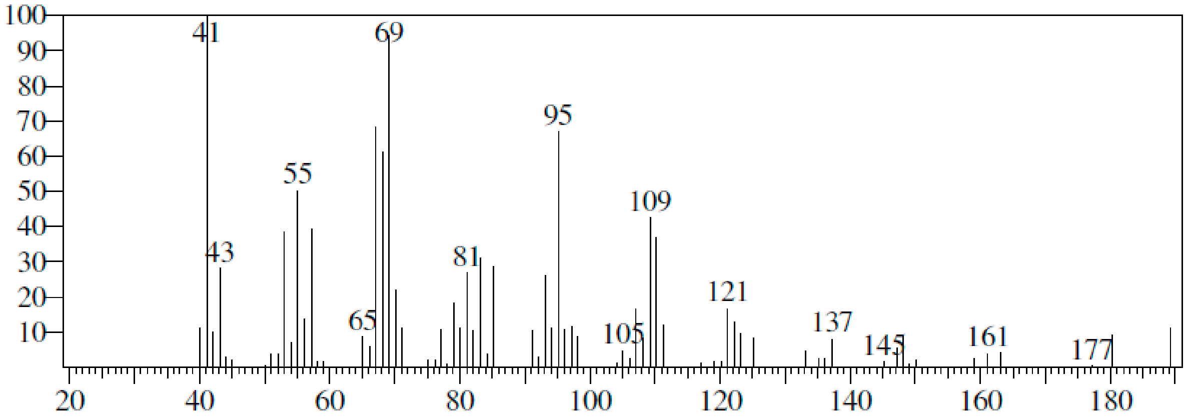

Peak 48

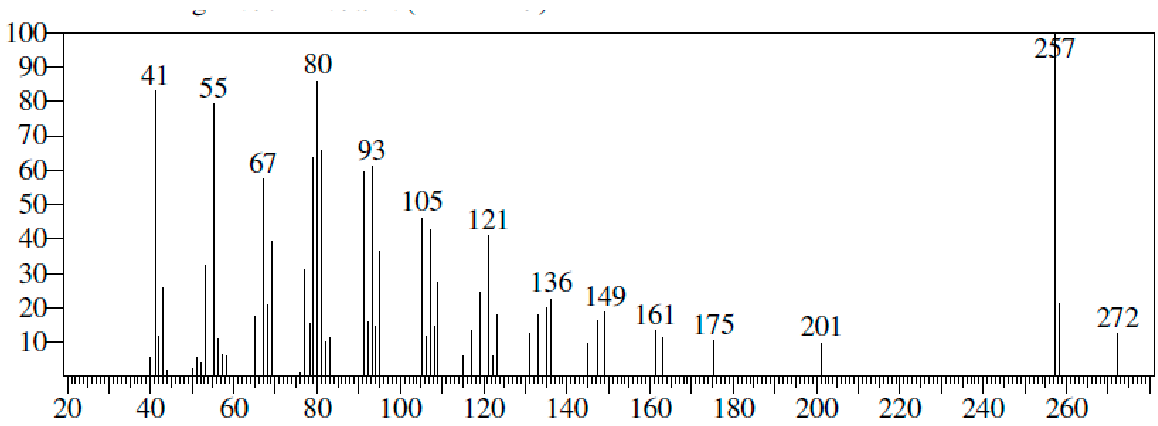

100
90
80
70
60
50
40
30
20
10
41
55
67
80
93
105
121
136
149
161
175
201
257
272
20
40
60
80
100
120
140
160
180
200
220
240
260
